# Lobar Pneumonia

**Published:** 1912-02

**Authors:** J. P. Liles

**Affiliations:** Roanoke, Ala.


### LOBAR PNUEMONIA.*

By J. P. Liles, M. D., Roanoke, Ala.

Lobar Pneumonia is an acute, infectious inflammatory disease of the lungs, characterized by high fever, rapid breathing, a full-bounding and quick pulse. The term Lobar Pneumonia is given to this type, because it involves at least a single lobe, or the greater part of it, and may involve all of them, which is rare.

A bacterium known as Diplococcus Pneumonia, Diplococcus Lanceolatus, is found in 75% of all cases, discovered by Frankle in 1886. While we consider Lobar Pneumonia as an infectious disease, yet we cannot ignore the operation of causes, such as cold and damp, lowered vitality and resisting powers; the causes, as we understand them to-day, are two-fold; namely: Predisposing and Exciting Causes; the predisposing causes, as are accepted are: exposure to cold and damp, lowered vital.ty and resisting force; the exciting cause is the bacillus of Frankle.

*Morbid Anatomy.*—This you are all too familiar with to take up your time in going over it, and I will only mention the stages:

1. Congestive or Engorgement. 2. Red Hepatization. 3. Gray Hepatization. Pneumonia is usually found in the lower lobes of the lungs, and more often in the right side, except in children, which, in my experience, is the reverse.

*Symptoms.*—At first a feeling of malaise, then a decided chill, followed almost immediately by fever from 103 to 105 with a full and strong pulse, almost incompressible, with a characteristic flush of the cheeks, as a rule more marked on the affected side; pulse 100 to 120 and often more; a pain develops in the side of varying severity, from dull pain to the most excruciating, from the involvement of the pleura. The respiration, rapid and painful. There is great thirst; the urine scanty and high-colored, and almost always albuminous. At first there is a small amount of mucous expectoration, aggravated cough. After the first 24 hours, we have the characteristic, tenacious, rusty sputum.

In this stage the respirations are extremely rapid, from 40 to 60 per minute. The physical symptoms I will not mention, for you are all too well informed to take up your time, nor will we consider the microscopic symptoms.

*Diagnosis.*—The diagnosis of a typical case of Pneumonia is easy. The chill, the rapidly developed fever, and the physical signs of it are, as a rule, easily recognized. It is to be remembered, however, that the physical signs may be delayed, or not appear at all in the central varieties. I will not go into the details, as I presume you all are too well-skilled as diagnosticians to consume your valuable time.

*Terminations.*—Resolutions are the most common.

Resolution may be unduly delayed, and yet ultimately take place, but such cases naturally cause anxiety. Resolution may not take place at all, in which one of the unfavorable terminations may occur:

1. Death from cardiac failure; this I have never seen.
2. Abscess; I have had only one in a practice of 14 years.
3. Gangrene; this I have never had.
4. Interstitial or Fibroid Pneumonia. 5. Tubercular Phthisis; neither of these have come under my observation.

*Complications.*—Pleurisy is the most common. It is always present to a certain extent, unless the involvement is of the central lobes.

Endocarditis is another complication to look for, and is common.

*Prognosis.*—The prognosis in every case of pneumonia should be guarded, as we know that it is a treacherous and uncertain disease at any age. The young and robust sometimes die suddenly and unexpectedly. In the old and temperate, it is especially dangerous, while the intemperate are less fortunate. Yet among them, some surprising recoveries are observed. Children recover often when desperately sick and even when recovery seems impossible. In my own practice, I have never had one to die over 3 months old. The disease seems to be more fatal in cities than in the country, and especially so during epidemics. The seriousness of an attacks depends, of course, largely upon the amount of lung involved, pneumonia of a whole lung being more dangerous than that of a part of a lung; double pneumonia more than that affecting one lung, while massive pneumonia is always fatal. Death is usually from heart failure.

*Treatment.*—In my experience, I would not dare to outline a single plan of treatment for pneumonia, each case being a law unto itself. This is true more or less of all diseases, but it is especially true of pneumonia. I find in the treatment of pneumonia, as I have in almost every serious disease, that the question with myself is, not what to give—but what not to give.

No doubt some cases would be greatly benefited by blood-letting, but I have never practiced it. I do not see but two periods in pneumonia where blood-letting would be advantageous:

1. In the first stage, or the beginning of the second stage. 2. When the right heart is engorged, the same results may be obtained by wet cups, although it is more painful and disagreeable to the patient. Ice to the side affected, is good treatment. But the most important of all the treatments is elimination. It matters not what else you do, if you fail to eliminate you have accomplished nothing, so far as benefiting your patient, for this purpose, no drug in the Materia Medica will do this as thoroughly as Calomel; to it may be added Powd. Ipecac, Padophlilin, and Bicarbonate of Soda, and give it until you get the results, regardless of the dose; then you have the dose. Repeat it as often as necessary during the course of the disease. Aside from the elimination from the calomel, it is the best defibrinator we have. Another never-failing drug in distress is Veratrum-Viride; this I give as I do calomel, not for its dose, but for its effect, while it is not indicated in every case of pneumonia, but where it is indicated there is no other drug that will take its place. The indication for its use is high fever, a full-bounding, rapid, incompressible pulse, not so much for the fever, but to control the pulse; and remember this, if you fail to control the pulse, you have failed in its use.

When Veratrum Viride is given give it in the beginning and do not wait until the latter stages to begin its use; here it may do great harm. Veratrum also is a good expectorant and should be given throughout the course when its use has been employed. Expectorants have their place, but not in the beginning. The ammonium salts come in here very nicely; of them all, I prefer ammonium carbonate. It is unquestionably the best liquifier, stimulant, and diuretic. Squills also in the expectorant stage work well. Stimulants must not be neglected, but they should be guarded, remembering that we must not over-stimulate a tired and worn-out heart; never begin stimulants in the beginning until it is an alcoholic or an old person. Alcohol, strychnine and Digitalis are the stimulants to be relied upon, but I have seen all of them do harm; either or all of them used judiciously and at the proper time will serve you well. Another drug that has served me well in the critical period is Nitroglycerine, but ordinarily it is not needed in the treatment of Pneumonia. There are still other drugs that have their places in the treatment of pneumonia; quinine and duotal are good drugs, and should be used throughout the course of the disease. For pain, opium and morphine are the best drugs to be used; Dovers' powders act well in many cases. Feeding is an important part of the treatment; milk and broths are all-sufficient. Another remedy I would not fail to mention is the old-fashioned fly blister, which so many have abused and criticized; in delayed resolution and many times in the beginning with a severe pleuritic pain, nothing acts better.

---

### SURGICAL SUGGESTIONS.

In case of primary hemorrhage, cut vessels which are not bleeding need not be ligated, provided the patient can be watched. When the vessel cannot be tied in the wound, ligation in continuity is permissible.

The treatment of varicose veins is not completed until the surgeon has discovered the constitutional causative factor and advised its elimination.

In removing extensive varicose veins, the surgeon should bear in mind that two operators can accomplish twice as much as one.

Better than temporary ligature of a large vessel is the application of a soft clamp which cannot damage its wall. In the absence of such a clamp an assistant may cause occlusive angulation by making gentle traction upon a ligature passed under the vessel.

—American Jorunal of Surgery.

